# A computationally efficient nonparametric approach for changepoint detection

**DOI:** 10.1007/s11222-016-9687-5

**Published:** 2016-07-28

**Authors:** Kaylea Haynes, Paul Fearnhead, Idris A. Eckley

**Affiliations:** 1 STOR-i Centre for Doctoral Training, Lancaster University, Lancaster, UK; 2 Department of Mathematics and Statistics, Lancaster University, Lancaster, UK

**Keywords:** Nonparametric maximum likelihood, PELT, CROPS, Activity tracking

## Abstract

In this paper we build on an approach proposed by Zou et al. (2014) for nonparametric changepoint detection. This approach defines the best segmentation for a data set as the one which minimises a penalised cost function, with the cost function defined in term of minus a non-parametric log-likelihood for data within each segment. Minimising this cost function is possible using dynamic programming, but their algorithm had a computational cost that is cubic in the length of the data set. To speed up computation, Zou et al. (2014) resorted to a screening procedure which means that the estimated segmentation is no longer guaranteed to be the global minimum of the cost function. We show that the screening procedure adversely affects the accuracy of the changepoint detection method, and show how a faster dynamic programming algorithm, pruned exact linear time (PELT) (Killick et al. 2012), can be used to find the optimal segmentation with a computational cost that can be close to linear in the amount of data. PELT requires a penalty to avoid under/over-fitting the model which can have a detrimental effect on the quality of the detected changepoints. To overcome this issue we use a relatively new method, changepoints over a range of penalties (Haynes et al. 2016), which finds all of the optimal segmentations for multiple penalty values over a continuous range. We apply our method to detect changes in heart-rate during physical activity.

**Electronic supplementary material:**

The online version of this article (doi:10.1007/s11222-016-9687-5) contains supplementary material, which is available to authorized users.

## Introduction

Changepoint detection is an area of statistics broadly studied across many disciplines such as acoustics (Guarnaccia et al. 2015; Lu and Zhang 2002), genomics (Olshen et al. 2004; Zhang and Siegmund 2007) and oceanography (Nam et al. 2014). Whilst the changepoint literature is vast, many existing methods are parametric. For example a common approach is to introduce a model for the data within a segment, use minus the maximum of the resulting log-likelihood to define a cost for a segment, and then define a cost of a segmentation as the sum of the costs for each of its segments. See for example Yao (1988), Lavielle (2005), Killick et al. (2012) and Davis et al. (2006). Finally, the segmentation of the data is obtained as the one that minimises a penalised version of this cost (see also Frick et al. 2014, for an extension of these approaches).

A second class of methods are based on tests for a single changepoint, with the tests often defined based on the type of change that is expected (such as change in mean), and the distribution of the null-statistic for each test depending on further modelling assumptions for the data (see e.g. Bai and Perron 1998; Dette and Wied 2015). Tests for detecting a single change can then be applied recursively to detect multiple changes, for example using binary segmentation (Scott and Knott 1974) or its variants (e.g. Fryzlewicz 2014). For a review of alternative approaches for change detection see Jandhyala et al. (2013) and Aue and Horvth (2013).

Much of the existing literature on nonparametric methods look at single changepoint detection (Page 1954; Bhattacharyya and Johnson 1968; Carlstein 1988; Dumbgen 1991). Several approaches are based on using rank statistics such as the Mann–Whitney test statistic (Pettitt 1979). Ross and Adams (2012) introduce the idea of using the Kolmogorov–Smirnov and the Cramer-von Mises test statistics; both of which use the empirical distribution function. Other methods include using kernel density estimations (Baron 2000), however these can be computationally expensive to calculate.

There is less literature on the nonparametric multiple changepoint setting. The single changepoint detection methods which have been developed using nonparametric methods do not extend easily to multiple changepoints. Within the sequential changepoint detection literature one can treat the problem as a single changepoint problem which resets every time a changepoint is detected (Ross and Adams 2012). Lee (1996) proposed a weighted empirical measure which is simple to use but has been shown to have unsatisfactory results. Under the multivariate setting Matteson and James (2014) and James and Matteson (2015) proposed methods, E-divisive and e-cp3o, based on clustering and probabilistic pruning respectively. The E-divisive method uses an exact test statistic with an approximate search algorithm whereas the e-cp3o method uses an approximate test statistic with an exact search algorithm. As a result e-cp3o is faster but lacks slightly in the quality for the changepoints detected.

In this article we focus on univariate changepoint detection and we are interested in the work of Zou et al. (2014) who propose a nonparametric likelihood based on the empirical distribution. They then use a dynamic programming approach, Segment Neighbourhood Search (Auger and Lawrence 1989), which is an exact search procedure, to find multiple changepoints. Whilst this method is shown to perform well, it has a computational cost of $\mathcal {O}(Mn^2+n^3)$ where *M* is the maximum number of changepoints and *n* is the length of the data. This makes this method infeasible when we have large data sets, particularly in situations where the number of changepoints increases with *n*. To overcome this, Zou et al. (2014) propose an additional screening step that prunes many possible changepoint locations. However, as we establish in this article, this screening step can adversely affect the accuracy of the final inferred segmentation.

In this paper we seek to develop a computationally efficient approach to the multiple changepoint search problem in the nonparametric setting. Our approach is an extension to the method of Zou et al. (2014), which uses the cumulative empirical distribution function to define segment costs. Our method firstly involves simplifying the definition of the segment cost, so that calculating the cost for a given segment involves computation that is $O(\log n)$ rather than *O*(*n*). Secondly we apply a different dynamic programming approach, pruned exact linear time (PELT) (Killick et al. 2012), that is substantially quicker than Segment Neighbourhood Search; for many situations where the number of changepoints increases linearly with *n*, PELT has been proven to have a computational cost that is linear in *n*.

We call the new algorithm ED-PELT, referring to the fact we have adapted PELT with a cost function based on the empirical distribution. A disadvantage of ED-PELT is that it requires the user to pre-specify a value by which the addition of a changepoint is penalised. The quality of the final segmentation can be sensitive to this choice, and whilst there are default choices these do not always work well. However we show that the Changepoints for a Range of PenaltieS (CROPS) algorithm (Haynes et al. 2016) can be used with ED-PELT to explore optimal segmentations for a range of penalties.

The rest of this paper is organised as follows. In Sect. 2 we give details of the NMCD approach proposed by Zou et al. (2014). In Sect. 3 we introduce our new efficient nonparametric search approach, ED-PELT, and show how we can substantially improve the computational cost of this method. In Sect. 4 we demonstrate the performance of our method on simulated data sets comparing our method with NMCD. Finally in Sect. 5 we include some simulations which analyse the performance of NMCD for different scenarios and then we show how a nonparametric cost function can be beneficial in situations where we do not know the underlying distribution of the data. In order to demonstrate our method we use heart-rate data recorded whilst an individual is running.

## Nonparametric changepoint detection

### Model

The model that we refer to throughout this paper is as follows. Assume that we have data, $x_1,...,x_n \in \mathbb {R}$ , that have been ordered based on some covariate information such as time or position along a chromosome. For $v\ge u$ we denote $x_{u:v} = \{x_{u},...,x_v\}$. Throughout we let *m* be the number of changepoints, and the positions be $\tau _1,\ldots ,\tau _m$. Furthermore we assume that $\tau _i$ is an integer and that $0 = \tau _0< \tau _1< \tau _2< ...< \tau _m < \tau _{m+1} = n$. Thus our *m* changepoints split the data into $m+1$ segments, with the *i*th segment containing $x_{\tau _{i-1}+1:\tau _{i}}$.

As in Zou et al. (2014) we will let $F_i(t)$ be the (unknown) cumulative distribution function (CDF) for the *i*th segment, and $\hat{F}_i(t)$ the empirical CDF. In other words

2.1 $$\begin{aligned} \hat{F}_i(t) {=}\frac{1}{\tau _i-\tau _{i-1}} \times \left( \sum _{j=\tau _{i-1}+1}^{\tau _i} \mathbf {1}\{x_j < t\} + 0.5 \times \mathbf {1}\{x_j {=} t\} \right) . \end{aligned}$$

Finally we let $\hat{F}(t)$ be the empirical CDF for the full data set.

### Nonparametric maximum likelihood

If we have *n* data points that are independent and identically distributed with CDF *F*(*t*), then, for a fixed value of *t*, the empirical CDF will satisfy $n\hat{F}(t)\sim \text{ Binomial }(n,F(t))$. Hence the log-likelihood of *F*(*t*) is given by: $n\{\hat{F}(t) \log ({F}(t)) + (1 - \hat{F}(t)) \log (1-{F}(t))\}.$ This log-likelihood is maximised by the value of the empirical CDF, $\hat{F}(t)$. We can thus use minus the maximum value of this log-likelihood as a segment cost function. So for segment *i* we have a cost that is $-\mathcal {L}_{np}(x_{\tau _{i-1}+1:\tau _i}|t)$ where

2.2 $$\begin{aligned}&\mathcal {L}_{np}(x_{\tau _{i-1}+1:\tau _i}|t) = (\tau _{i} - \tau _{i-1})\times [\hat{F}_i(t) \log \hat{F}_i(t) \nonumber \\&\qquad \qquad \qquad \qquad \quad +\,(1-\hat{F}_i(t)) \log (1-\hat{F}_i(t))]. \end{aligned}$$

We can then define a cost of a segmentation as the sum of the segment costs. Thus to segment the data with *m* changepoints we minimise $-\sum _{i=1}^{m+1}\mathcal {L}_{np}(x_{\tau _{i-1}+1:\tau _i}|t)$.

### Nonparametric multiple changepoint detection

One problem with the segment cost as defined by (2.2) is that it only uses information about the CDF evaluated at one value of *t* and that the choice of *t* can have detrimental effects on the resulting segmentations. To overcome this Zou et al. (2014) suggest defining a segment cost which integrates (2.2) over different values of *t*. They suggest a cost function for a segment with data $x_{u:v}$ that is

2.3 $$\begin{aligned} \int _{-\infty }^{\infty } -\mathcal {L}_{np}(x_{u:v}|t) dw(t), \end{aligned}$$

with a weight, $dw(t) = \{{F}(t)(1-{F}(t))\}^{-1} d{F}(t)$, that depends on the CDF of the full data. This weight is chosen to produce a powerful goodness of fit test (Zhang 2002). As this is unknown they approximate it by the empirical CDF of the full data, and then further approximate the integral by a sum over the data points. This gives the following objective function

2.4 $$\begin{aligned}&Q_{\text {NMCD}}(\tau _{1:m}|x_{1:n})= -n\sum _{i=1}^{m+1} \sum _{t = 1}^{n}(\tau _{i} - \tau _{i-1}) \nonumber \\&\quad \times \frac{\hat{F}_i(t) \log \hat{F}_i(t) + (1-\hat{F}_i(t))\log (1-\hat{F}_i(t))}{(t - 0.5)(n-t+0.5)}. \end{aligned}$$

For a fixed *m* this objective function is minimised to find the optimal segmentation of the data.

In practice a suitable choice of *m* is unknown, and Zou et al. (2014) suggest estimating *m* using the Bayesian Information criterion (Schwarz 1978). That is, they minimise

2.5 $$\begin{aligned} \text {BIC} = \min _{m|\tau _1,...,\tau _m} \left\{ Q_{\text {NMCD}}(\tau _{1:m}|x_{1:n}) + m \xi _n\right\} , \end{aligned}$$

where $\xi _n$ is a sequence going to infinity.

### NMCD algorithm

To maximise the objective function (2.4), Zou et al. (2014) use the dynamic programming algorithm Segment Neighbourhood Search (Auger and Lawrence 1989). This algorithm calculates the optimal segmentations, given a cost function, for each value of $m=1,\ldots ,M$, where *M* is a specified maximum number of changepoints to search for. If all the segment costs have been pre-computed then Segment Neighbourhood search has a computational cost of $\mathcal {O}(Mn^2)$. However for NMCD the segment cost involves calculating

$$\begin{aligned} \sum _{t = 1}^{n}\frac{\hat{F}_i(t) \log \hat{F}_i(t) + (1-\hat{F}_i(t))\log (1-\hat{F}_i(t))}{(t - 0.5)(n-t+0.5)}, \end{aligned}$$

and thus calculating the cost for a single segment is *O*(*n*). Hence the cost of precomputing all segment costs is $O(n^3)$, and the resulting algorithm has a cost that is $O(Mn^2+n^3)$.

To reduce the computational burden when we have long data series, Zou et al. (2014) propose a screening step. They consider overlapping windows of length $2N_I$ for some $N_I \in \mathbb {R}$. For each window they calculate the Cramér-von Mises (CvM) statistic for a changepoint at the centre of the window. They then compare these CvM statistics, each corresponding to a different changepoint location, and remove a location as a candidate changepoint if its CvM statistic is smaller than any of the CvM statistics for locations within $N_I$ of it. The number of remaining candidate changepoint positions is normally much smaller than *n* and thus the computational complexity can be substantially reduced. The choice of $N_I$ is obviously important, with larger values leading to the removal of more putative changepoint locations, but at the risk or removing true changepoint locations. In particular, the rationale for the method is based on $N_I$ being smaller than any segment that you wish to detect. As a default, Zou et al. (2014) recommend choosing $N_I = \lceil (\log n)^{3/2}/2 \rceil$ where $\lceil x \rceil$ denotes the smallest integer which is larger than *x*.

## ED-PELT

Here we develop a new, computationally efficient, way to segment data using a cost function based on (2.3). This involves firstly an alternative numerical approximation to the integral (2.3), which is more efficient to calculate. In addition we use a more efficient dynamic programming algorithm, PELT (Killick et al. 2012), to then minimise the cost function.

### Discrete approximation

To reduce the cost of calculating the segment cost, we approximate the integral by a sum with $K<<n$ terms. The integral in (2.3) involves a weight, and we first make a change of variables to remove this weight.

#### Lemma 3.1

Let $c=-\log (2n-1)$. For $z \in [-1,1]$ define $p(z)=(1+\exp \{cz\})^{-1}$. Then

3.1 $$\begin{aligned}&\int _{\frac{1}{2n}}^{\frac{2n-1}{2n}} \mathcal {L}_{np}(x_{u:v}|t) \{F(t)(1-F(t))\}^{-1} dF(t) \nonumber \\&\quad =-c \int _{-1}^1 \mathcal {L}_{np}(x_{u:v}|F^{-1}(p(z))) dz. \end{aligned}$$

#### Proof

This follows from making the change of variable $F(t)=p(z)$. $\square$

Using Lemma 3.1, we suggest the following approximation, based on an approximation of (3.1) using *K* unevenly spaced *x*-values. We choose these *x*-values specifically to give higher weight to values in the tail of the distribution of the data. Our approximation achieves this through a sum where each term has equal weight, but where the *x*-values we choose are preferentially chosen from the tail of the distribution. That is we fix *K*, and let $t_1,\ldots ,t_K$ be such that $t_k$ is the $(1+(2n-1)\exp \{\frac{c}{K}(2k-1)\})^{-1}$ empirical quantile of the data, where *c* is defined in Lemma 3.1. then we approximate (2.3) by

3.2 $$\begin{aligned} \mathcal {C}_K(x_{u:v})= \frac{-2c}{K} \sum _{k=1}^K \mathcal {L}_{np}(x_{u:v}|t_k). \end{aligned}$$

The cost now for calculating the segment costs is $\mathcal {O}(K)$. We show empirically in Sect. 4 that this choice of *K* can lead to segment costs of $\mathcal {O}(\log n)$.

### Use of PELT

We now turn to consider how the PELT approach of Killick et al. (2012) can be incorporated within this framework. The PELT dynamic programming algorithm is able to solve minimisation problems of the form

$$\begin{aligned} Q_{\text {PELT}}(x_{1:n}|\xi _n) = \min _{m,\tau _{1:m}} \left\{ \sum _{i=1}^{m+1}[ \mathcal {C}_K(x_{\tau _{i-1}+1:\tau _i}) + \xi _n] \right\} . \end{aligned}$$

It jointly minimises over both the number and position of the changepoints, but requires the prior choice of $\xi _n$, the penalty value for adding a changepoint. The PELT algorithm uses the fact that $Q_{\text {PELT}}(x_{1:n})$ is the solution of the recursion, for $v>1$

3.3 $$\begin{aligned} Q_{\text {PELT}}(x_{1:v}|\xi _n)=\min _{u< v}\left( Q_{\text {PELT}}(x_{1:u})+ \mathcal {C}_K(x_{u+1:v}) + \xi _n\right) . \end{aligned}$$

The interpretation of this is that the term in the brackets on the right-hand side of Eq. 3.3 is the cost for segmenting $x_{1:v}$ with the most recent changepoint at *u*. We then optimise over the location of this most recent changepoint. Solving the resulting set of recursions leads to an $O(n^2)$ algorithm (Jackson et al. 2005), as (3.3) needs to be solved for $v=2,\ldots ,n$; and solving (3.3) for a given value of *v* involves a minimisation over *v* terms.

The idea of PELT is that we can substantially speed up solving (3.3) for a given *v* by reducing the set of values of *u* we have to minimise over. This can be done through a simple rule that enables us to detect time points *u* which can never be the optimal location of the most recent changepoint at any subsequent time. For our application this comes from the following result

#### Theorem 3.2

If at time *v*, we have $u<v$ such that

3.4 $$\begin{aligned} Q_{\text {PELT}}(x_{1:u}\xi _n) + \mathcal {C}_K(x_{u+1:v}) \ge Q_{\text {PELT}}(x_{1:v}|\xi _n), \end{aligned}$$

then for any future time $T > v$, *u* can never be the time of the optimal last changepoint prior to *T*.

#### Proof

This follows from Theorem 3.1 of Killick et al. (2012), providing we can show that for any $u<v<T$

3.5 $$\begin{aligned} \mathcal {C}_K(x_{u+1:T}) \ge \mathcal {C}_K(x_{u+1:v})+\mathcal {C}_K(x_{v+1:T}). \end{aligned}$$

As $\mathcal {C}_K(\cdot )$ is a sum of *k* terms, each of the form $-\mathcal {L}_{np}(\cdot |t_k)$ we need only show that for any *t*

$$\begin{aligned} \mathcal {L}_{np}(x_{u+1:T}|t) \le \mathcal {L}_{np}(x_{u+1:v}|t)+\mathcal {L}_{np}(x_{v+1:T}|t). \end{aligned}$$

Now if we introduce notation that $\hat{F}_{u,v}(t)$ is the empirical CDF for data $x_{u:v}$, we have

$$\begin{aligned} \mathcal {L}_{np}(x_{u+1:T}|t)&=(T-u)[\hat{F}_{u,T}(t)\log (\hat{F}_{u,T}(t))\\&\quad +(1-\hat{F}_{u,T}(t))\log (1-\hat{F}_{u,T}(t))]\\&=\{(v-u)[\hat{F}_{u,v}(t)\log (\hat{F}_{u,T}(t))\\&\quad +(1-\hat{F}_{u,v}(t))\log (1-\hat{F}_{u,T}(t))] \\&\quad + (T-v)[\hat{F}_{v,T}(t)\log (\hat{F}_{u,T}(t))\\&\quad +(1-\hat{F}_{v,T}(t))\log (1-\hat{F}_{u,T}(t))] \}\\&\le \mathcal {L}_{np}(x_{u+1:v}|t)+\mathcal {L}_{np}(x_{v+1:T}|t), \end{aligned}$$

as required. $$\square $$

Thus at each time-point we can check whether (3.4) holds, and if so prune time-point *u*. Under certain regularity conditions, Killick et al. (2012) show that for models where the number of changepoints increases linearly with *n*, such substantial pruning occurs that the PELT algorithm will have an expected computational cost that is *O*(*n*). We call the resulting algorithm we obtain ED-PELT (PELT with a cost based on the empirical distribution).

## Results

### Performance of NMCD

We firstly compare the NMCD algorithm with (NMCD+) and without screening (NMCD) using the nmcdr R package (Zou and Zhange (2014)), with the default choices $\xi _n$ (Bayesian Information Criterion) and in the NMCD+ algorithm $N_I$ as detailed in Section 2.4. We set up a similar simulation as in Zou et al. (2014). That is, we simulate data of length $n=1000$ from the following three models, where $J(x) = \{1+sgn(x)\}/2$.

*Model 1:* $$x_i = \sum _{j=1}^{M} h_j J(nt_i - \tau _j) + \sigma \xi _i$$, where

$$\begin{aligned}&\{\tau _j/n\} = \{0.1, 0.13, 0.15, 0.23, 0.25, 0.40, 0.44,\\&\qquad \qquad \quad 0.65, 0.76, 0.78, 0.81\}, \\&\{h_j\} = \{2.01, -2.51, 1.51, -2.01, 2.51, \\&\qquad \qquad -2.11, 1.05, 2.16, -1.56, 2.56, -2.11\}, \end{aligned}$$

and there are *n* equally spaced $t_i$ in [0, 1]. *Model 2:* $x_i = \sum _{j=1}^{M} h_i J(nt_i - \tau _j) + \sigma \xi _i \prod _{j=1}^{\sum _{j=1}^{M}J(nt_i - \tau _j)} v_j$, where

$$\begin{aligned}&\{\tau _j/n\} = \{0.20, 0.40, 0.65, 0.85\}, \{h_j\} = \{3,0,-2,0\},\\&\quad \text {and} \; \{v_j\} = \{1,5,1,0.25\}. \end{aligned}$$

*Model 3:* $x_i \sim F_j(x)$, where $\tau _j/n = \{0.20, 0.50, 0.75\}$, $j = 1,2,3,4$, and $F_1(x),...,F_4(x)$ corresponds to the standard normal, the standardized $\chi _{(3)}^2$ (with zero mean and unit variance), the standardized $\chi _{(1)}^2$ and the standard normal distribution respectively.

The first model has $M=11$ changepoints, all of which are changes in location. Model 2 has both changes in location and in scale and model 3 has changes in skewness and in kurtosis. For the first two models we also consider three distributions for the error, $\xi _i$: *N*(0, 1), Student’s *t* distribution with 3 degrees of freedom and the standardised chi-square distribution with one degree of freedom, $\chi _{(1)}^2$.

To compare both the NMCD and NMCD+ we first look at the true and false discovery rates. That is a detected changepoint $\hat{\tau }_i$ is true if $\min _{1 \le j \le m} \{|\hat{\tau }_i - \tau _j|\} \le h$, where *m* is the true number of changepoints and *h* is some threshold. In this case we will use $h=0$. That is a detected changepoints is only counted as true if it is in the correct location. The number of true detected changepoints is thus $\hat{m}_{\text {TRUE}} = \sum _{i=1}^{\hat{m}}\mathbbm {1}_{\min _{1 \le j \le m} \{|\hat{\tau }_i - \tau _j|\} \le 0}$, where $\hat{m}$ is the number of detected changepoints. The true discovery rate (TDR) and false discovery rate (FDR) are then calculated as:

4.1 $$\begin{aligned} \text {TDR} = \frac{\hat{m}_{\text {TRUE}}}{m}, \quad \text {FDR} = \frac{\hat{m}_{\text {FALSE}}}{\hat{m}} = \frac{1-\hat{m}_{\text {TRUE}}}{\hat{m}}. \end{aligned}$$

**Table 1:** True and false discovery rates and time comparisons for NCMD, NMCD+ and ED-PELT. Values in the table are mean (standard errors in parentheses) for 100 replications

	$\text {True Discovery Rate}$			$\text {False Discovery Rate}$			Time		
	NMCD	NMCD+	ED-PELT	NMCD	NMCD+	ED-PELT	NMCD (min)	NMCD+ (s)	ED-PELT (s)
(I)									
*N*(0, 1)	0.927 (0.002)	0.912 (0.003)	0.924 (0.002)	0.073 (0.002)	0.088 (0.003)	0.076 (0.002)	19.403 (0.051)	1.677 (0.007)	0.102 (0.003)
$t_{(3)}$	0.803 (0.004)	0.763 (0.004)	0.796 (0.004)	0.233 (0.004)	0.240 (0.004)	0.210 (0.004)	21.037 (0.065)	1.685 (0.008)	0.129 (0.003)
$\chi ^2_{(3)}$	0.908 (0.003)	0.834 (0.003)	0.911 (0.003)	0.097 (0.003)	0.166 (0.003)	0.091 (0.003)	19.623 (0.040)	1.651 (0.006)	0.159 (0.001)
(II)									
*N*(0, 1)	0.580 (0.007)	0.398 (0.005)	0.583 (0.007)	0.454 (0.007)	0.609 (0.005)	0.424 (0.007)	19.963 (0.061)	1.577 (0.002)	0.288 (0.006)
$t_{(3)}$	0.482 (0.006)	0.323 (0.005)	0.487 (0.006)	0.567 (0.006)	0.695 (0.005)	0.527 (0.006)	10.732 (0.088)	1.578 (0.001)	0.216 (0.002)
$\chi ^2_{(3)}$	0.492 (0.006)	0.390 (0.005)	0.502 (0.006)	0.513 (0.007)	0.612 (0.005)	0.498 (0.006)	22.113 (0.050)	1.638 (0.002)	0.317 (0.002)
(III)									
	0.477 (0.008)	0.363 (0.008)	0.477 (0.002)	0.531 (0.008)	0.640 (0.008)	0.524 (0.002)	24.717 (0.014)	1.516 (0.015)	0.351 (0.002)

**Table 2:** Over-segmentation and under-segmentation errors, and the number of changepoints detected for NCMD, NMCD+ and ED-PELT. Values in the table are mean (standard errors in parentheses) for 100 replications

	Over Segmentation error			Under Segmentation error			Number of detected changepoints		
	NMCD	NMCD+	ED-PELT	NMCD	NMCD+	ED-PELT	NMCD	NMCD+	ED-PELT
(I)									
*N*(0, 1)	1.080 (0.055)	1.170 (0.042)	1.280 (0.063)	1.080 (0.055)	1.170 (0.042)	1.280 (0.063)	11.000 (0.000)	11.000 (0.000)	11.000 (0.000)
$t_{(3)}$	2.850 (0.096)	3.120 (0.097)	2.530 (0.077)	14.420 (0.822)	5.000 (0.382)	2.860 (0.093)	11.560 (0.091)	11.040 (0.020)	11.100 (0.036)
$\chi ^2_{(3)}$	0.970 (0.029)	2.290 (0.059)	0.990 (0.032)	2.490 (0.343)	2.290 (0.059)	1.030 (0.033)	11.070 (0.033)	11.000 (0.000)	11.030 (0.017)
(II)									
*N*(0, 1)	5.160 (0.194)	8.660 (0.217)	4.860 (0.175)	13.680 (0.768)	12.080 (0.494)	5.780 (0.269)	4.290 (0.057)	4.090 (0.032)	4.060 (0.028)
$t_{(3)}$	9.940 (0.286)	12.760 (0.267)	10.980 (0.354)	23.830 (0.896)	23.450 (0.764)	16.280 (0.622)	4.590 (0.091)	4.300 (0.063)	4.160 (0.047)
$\chi ^2_{(3)}$	6.800 (0.242)	9.630 (0.247)	7.090 (0.238)	7.730 (0.317)	10.310 (0.320)	7.090 (0.238)	4.070 (0.029)	4.010 (0.010)	4.000 (0.000)
(III)									
	2.730 (0.076)	5.730 (0.152)	3.030 (0.104)	4.730 (0.386)	6.000 (0.165)	3.240 (0.121)	3.060 (0.028)	3.020 (0.014)	3.010 (0.010)

It is clear from Table 1 that using the screening step (NMCD+) significantly improves the computational cost of NMCD. However using this screening step comes at a cost of not correctly detecting the true changepoints. It can be seen that in all cases NMCD+ detects fewer true positives and more false positives than NMCD.

These measures provide a good evaluation of the number as well as location of changepoints. In order to explore the accuracy of the changepoint locations further we can use the distance measures as in Zou et al. (2014). That is we can use the worst case distance between the true changepoint set and the false changepoint set as in Boysen et al. (2009). If we set $\varvec{\tau }$ as the set of true changepoints and $\hat{\varvec{\tau }}$ as the set of detected changepoints then the over-segmentation and under-segmentation error are calculated, respectively, as:

4.2 $$\begin{aligned} d(\hat{\varvec{\tau }},\varvec{\tau })&= \max _{1\le i \le \hat{m}} \min _{1\le j \le m} |\hat{\tau }_i - \tau _j| \quad \text {and}\nonumber \\ d(\varvec{\tau },\hat{\varvec{\tau }})&= \max _{1\le j \le m} \min _{1\le i \le \hat{m}} |\tau _j - \hat{\tau }_i|. \end{aligned}$$

Table 2 gives the average of over-segmentation and under-segmentation error for NMCD and NMCD+ as well as the number of detected changepoints. The over segmentation error is higher for NMCD+ than it is for NMCD in all models. In model 1 with the Normal errors both NMCD and NMCD+ found the same number of changepoints for all models but on average NMCD was more accurate than NMCD+. The under segmentation error is comparable for both NMCD and NMCD+ for all models except model 1 with Student’s *t* distribution error where the under segmentation error is much higher for NMCD than NMCD+ and in model 2 with chi-squared error where the under-segmentation error is much higher for NMCD+ than NMCD. In all cases NMCD+ found the same or less number of changepoints than NMCD but closer to the true number. However even though NMCD+ detected the true number of changepoints more we see that the locations of these changepoints were most of the time less accurate than NMCD.

### Size of screening window

**Fig. 1:**
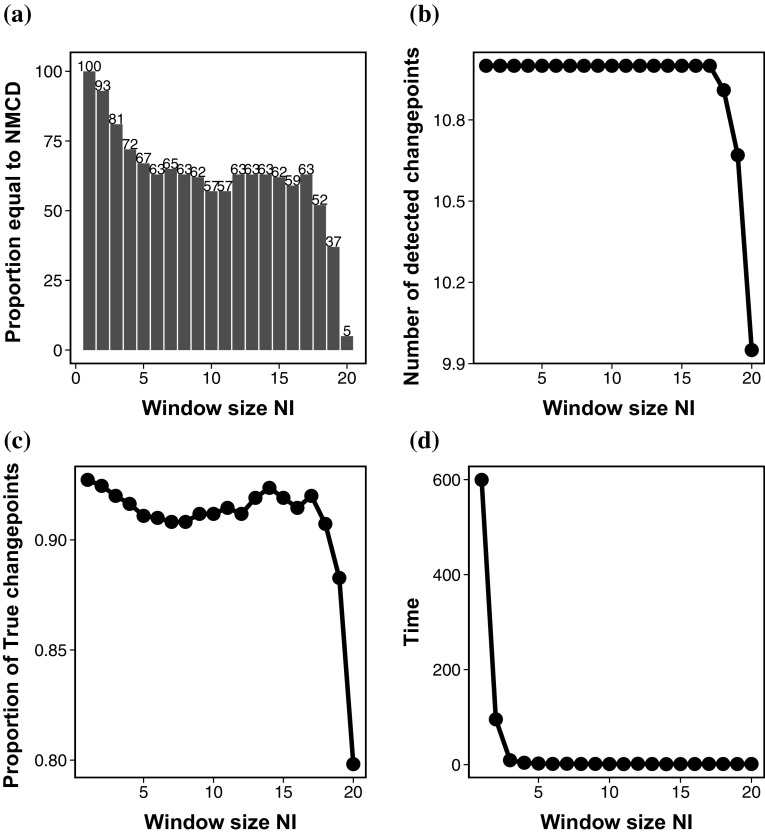
**a** The number of replications out of 100 in which using NMCD+ with varying $N_I$ results in the same results as NMCD without screening. **b** The number of changepoints detected with with increasing window size $N_I$. **c** The proportion of true changepoints detected with varying window size $N_I$. **d** The computational time (s) for NMCD+ with increasing window size $N_I$

We now turn to consider the choice for the size of the screening window $N_I$ further. Using Model 1 with normal errors we can compare the results for different values of $N_I$. The default value for this data is $N_I=10$, but we now repeat the analysis using $N_I \in \{1,\ldots ,20\}$. Figure 1a shows a bar plot of the number of times (in 100 simulations) that the window size resulted in the same changepoints as using NMCD without screening. Figure 1b shows the number of changepoints detected with different window lengths and Fig. 1c looks at the number of true and false positives found using the different window lengths in the screening step. Figure 1d shows the computational time taken for NMCD+ with varying window lengths $N_I$. We found similar results for the other models.

**Fig. 2:**
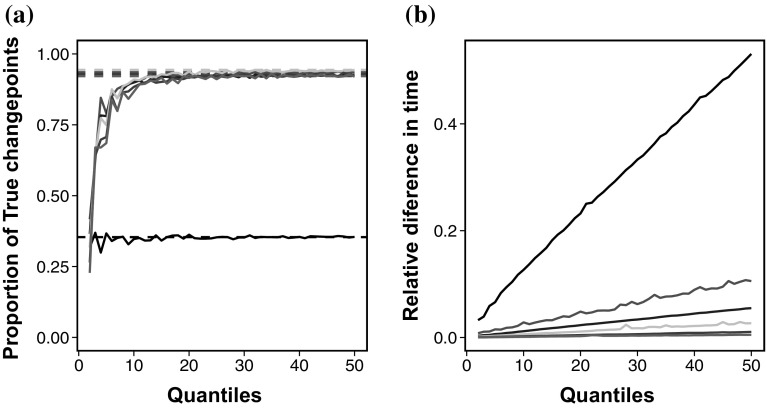
**a** The proportion of true positive changepoints for a range of quantiles, *K*, in ED-PELT (*solid*) in comparison to ED-PELT (*dashed*). *Black* $n = 100$, *red* $n = 500$, *blue*: $n = 1000$, *grey* $n = 2000$ and *dark green* $n = 5000$. **b** Relative speed of using ED-PELT compared to using ED-PELT. with varying number of quantiles, *K*. *Black* $n = 100$, *red* $n = 500$, *blue* $n = 1000$ , *grey* $n = 3000$, *dark green* $n = 5000$ and *purple* *n* =10,000. (Color figure online)

It is clear that whilst in the majority of the cases NMCD+ with the different $N_I$ find the correct number of changepoints the location of these are not always correct and in fact are different than that found using NMCD. It is also worth noting that even though many window sizes find 11 changepoints the location of these may be different for different window lengths. In general the performance decreases as window length increases however the results do fluctuate a bit. This shows that the performance of NMCD+ is sensitive to the choice of the window size. Despite this we can see that NMCD+ is significantly faster than NMCD especially as the window length increases.

The NMCD method also requires us to choose a penalty value in order to pick the best segmentation. The default choice appears to work reasonably well, but resulted in slight over-estimates of the number of changepoints for our three simulation scenarios. These over-estimates suggest that the penalty value has been too small.

### Choice of *K* in ED-PELT

We now turn our attention to ED-PELT. In order to use the improvement suggested in Sect. 3.1 for ED-PELT we first of all need to decide on an appropriate value for *K*. We use Model 1 again to assess the performance of ED-PELT using only *K* quantiles of the data (ED-PELT), for a range of values of *K*, in comparison to ED-PELT using the full data set. Here we only look at the model with normal errors and simulate data-series with lengths $n = (100,500,1000,2000,5000,10000)$. Further simulations using different error terms gave similar results. In order to assess performance we look at the proportion of true positives detected using both methods and also the computational cost. Again we use 100 replications. The results for the accuracy can be seen in Fig. 2a.

We can see from Fig. 2a that as the number of quantiles increases the proportion of true change points detected using ED-PELT converges to the same result as ED-PELT. As the length of the data increases this convergence appears to happen more slowly, this can be seen from the purple line in Fig. 2a, which represents data of length 10000. We suggest using $K =\lceil 4\log (n) \rceil$ in order to conserve as much accuracy as possible. This choice corresponds to $K=19, 25$, 28, 31, 35 and 37 for $n = 100, 500$, 1000, 2000, 5000 and 10,000 respectively.

In addition to the accuracy we also look at the relative speed up of ED-PELT with various *K* values in comparison to ED-PELT, i.e.,

$$\begin{aligned} \frac{\text{(speed } \text{ of } \text{ ED-PELT) }}{\text{ speed } \text{ of } \text{ ED-PELT }}. \end{aligned}$$

The results of this analysis can be seen in Fig. 2b. Clearly as the number of quantiles increases the relative speed up decreases. This is expected since the number of quantiles is converging to the whole data set which is used in ED-PELT. We can also see that the relative speed up of ED-PELT increases with increasing data length.

### Comparison of NMCD and ED-PELT

We next compare ED-PELT with $K = 4\log (n)$ to NMCD as above. For this we perform an equivalent analysis to that of Sect. 4.1 and again look at the accuracy of the methods and the computational time. As before, to implement NMCD we used the nmcdr R package (Zou and Zhange 2014) which is written in FORTRAN with an R user interface. We use the changepoint.np R package (Haynes 2016) to run ED-PELT which also has an R interface but with the main body of the code written in C. We use the default parameters for nmcd and for ED-PELT we use the SIC/BIC penalty term, $2p \log (n)$, where *p* is the number of parameters, to match the penalty term used in the nmcd algorithm.

The results for ED-PELT can be found in Tables 1 and 2. In terms of accuracy we can see that ED-PELT is comparable to NMCD albeit lacking slightly in some of the measures, however it is significantly faster to run. We can also see from table 2 that ED-PELT has lower under-segmentation error than NMCD in most of the models, however it has a higher segmentation error. In comparison to NMCD+, EP-PELT+ is faster and also more accurate so would be the better approximate method to choose.

## Activity tracking

In this section we apply ED-PELT to try to detect changes in heart-rate during a run. Wearable activity trackers are becoming increasingly popular devices used to record step count, distances (based on the step count), sleep patterns and in some of the newer devices, such as the Fitbit change HR (Fitbit Inc., San Francisco, CA), heart-rate. The idea behind these devices is that the ability to monitor your activity should help you lead a fit and active lifestyle. Changepoint detection can be used in daily activity tracking data to segment the day into periods of activity, rest and sleep.

Similarly, many keen athletes, both professional and amateur, also use GPS sports watches which have the additional features of recording distance and speed which can be very beneficial in training, especially in sports such as running and cycling. Heart-rate monitoring during training can help make sure you are training hard enough without over training and burning out. Heart-rate is the number of heart beats per unit time, normally we express this as beats per minute (bpm).

### Changepoints in heart-rate data

In the changepoint and signal processing literature many authors have looked at heart-rate monitoring in different scenarios (see for example Khalfa et al. (2012); Galway et al. (2011); Billat et al. (2009); Staudacher et al. (2005)). Aubert et al. (2003) give a detailed review of the influence of heart-rate variability in athletes. They highlight the difficulty of analysing heart-rate measurements during exercise since no steady state is obtained due to the heart-rate variability increasing according to the intensity of the exercise. They note that one possible solution is to pre-process the data to remove the trend.

In this section we apply ED-PELT to see whether changes can be detected in the raw heart-rate time series without having to initially pre-process the data. We use a nonparametric approach since heart-rate is a stochastic time dependent series and thus does not satisfy the conditions for an IID Normal model. However we will compare the performance had we assumed that the data was Normal in Sect. 5.3. The aim is to develop a method which can be used on data recorded from commercially available devices without the need to pre-process the data.

### Range of penalties

One disadvantage of ED-PELT over NMCD is that ED-PELT produces a single segmentation, which is optimal for the pre-chosen penalty value $\xi _n$. By comparison, NCMD finds a range of segmentations, one for each of $m=1,\ldots ,M$ changepoints (though, in practice, the nmcdr package only outputs a single segmentation). Whilst there are default choices for $\xi _n$, these do not always work well especially in real-life applications where the assumptions they are based on do not hold. There are also advantages to being able to compare segmentations with different number of changepoints.

**Fig. 3:**
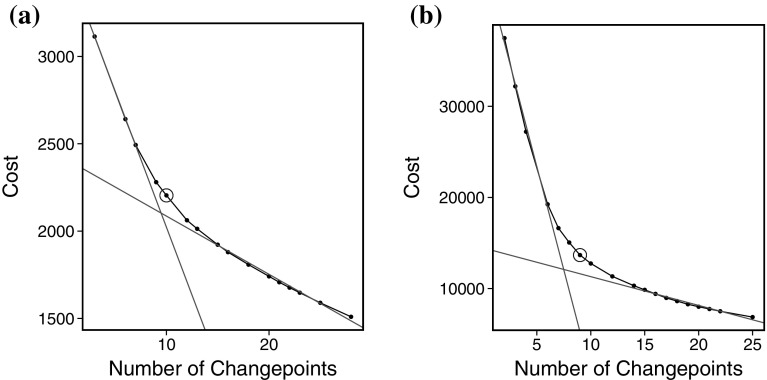
The cost vs number of changepoints plotted for **a** ED-PELT and **b** Change in slope. The *red lines* indicate the elbow and the *blue circle* highlights the point that we use as being the centre of the elbow. (Color figure online)

**Fig. 4:**
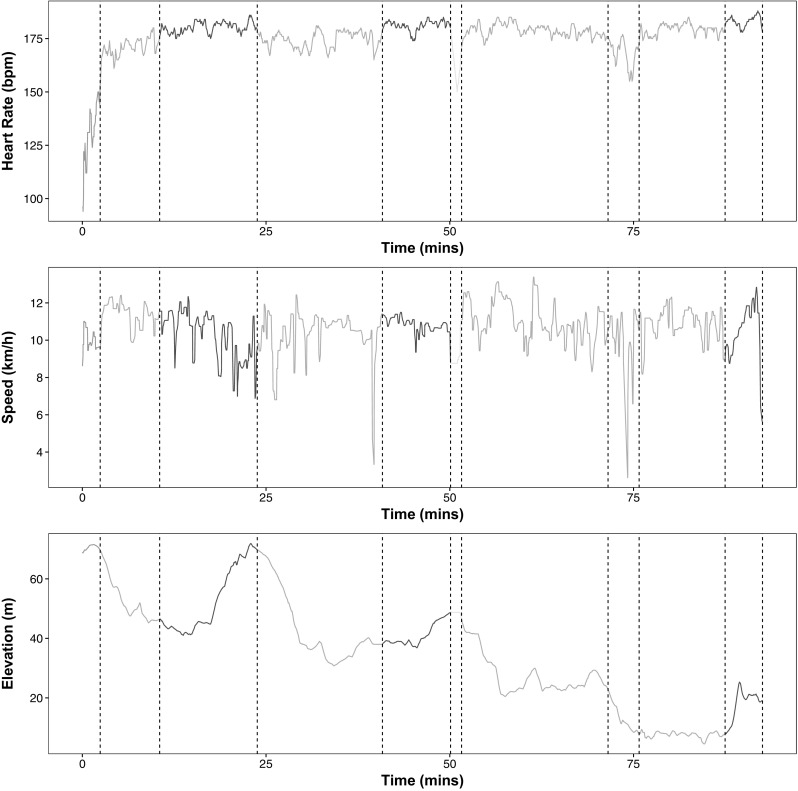
Segmentations using ED-PELT with 10 changepoints. We have colour coded the line based on the average heart-rate of each segment where *red* peak, *orange* anaerobic, *yellow* aerobic and *green* recovery. (Color figure online)

Haynes et al. (2016) propose a method, Changepoints over a Range Of PenaltieS (CROPS), which efficiently finds all the optimal segmentations for penalty values across a continuous range. This involves an iterative procedure which chooses values of $\xi _n$ to run ED-PELT on, based on the segmentations obtained from previous runs of ED-PELT for different penalty values. Assume we have a given range $[\xi _{\min },\xi _{\max }]$ for the penalty value, and the optimal segmentations at $\xi _{\min }$ and $\xi _{\max }$ have $m_{\min }$ and $m_{\max }$ changepoints respectively. Then CROPS requires at most $m_{\min }-m_{\max }+2$ runs of ED-PELT to be guaranteed to find all optimal segmentations for $\xi _n \in [\xi _{\min },\xi _{\max }]$. Furthermore, it is possible to recycle many of the calculations from early runs of ED-PELT to speed up the later runs.

#### Nonparametric changepoint detection

An example data set is given in Fig. 4, where we show heart-rate, speed and elevation recorded during a 10 mile run. We will aim to segment this data using the heart-rate data only, but include the other two series in order that we may assess how well the segmentation of the heart-rate data relates to the obvious different phases of the run. As is common in nonparametric methods, ED-PELT assumes that data is IID which in the case of heart-rate data the assumptions do not hold since there is some time-series dependence between segments. However for the moment we will assume all the assumptions hold and that we can use this method. In training many people use heart-rate as an indicator of how hard they are working. There are different heart-rate zones that you can train in each of which enhances different aspects of your fitness (BrainMacSportsCoach 2015). The training zones are defined in terms of percentages of a maximum heart-rate: peak (90–100 $\%$), anaerobic (80–90 $\%$), aerobic (70–80 $\%$) and recovery (<70 %).

**Fig. 5:**
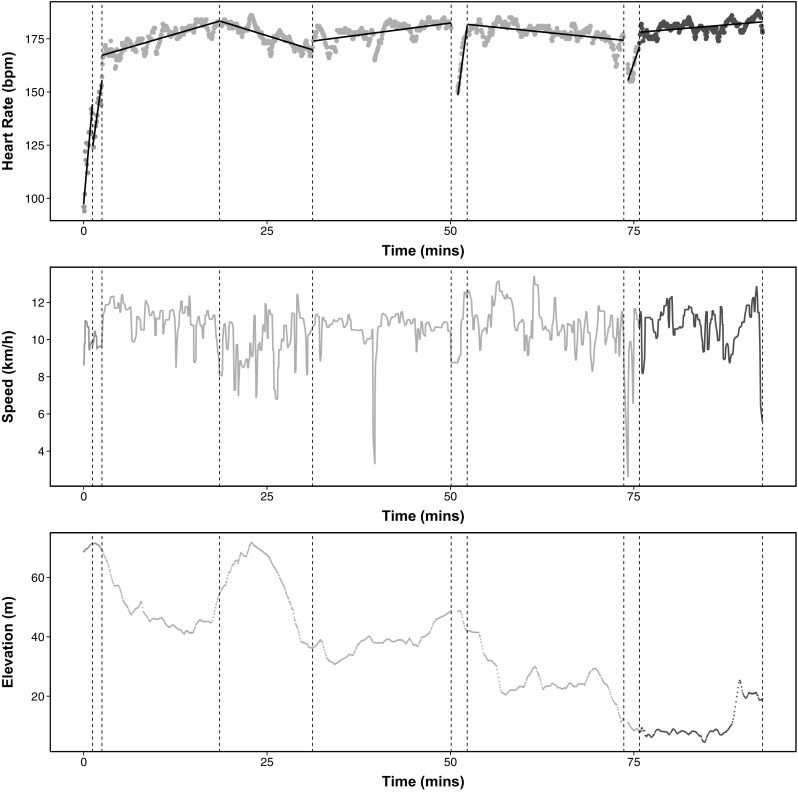
Segmentations using change in slope with 9 changepoints. We have colour coded the line based on the average heart-rate of each segment where *red* peak, *orange* anaerobic, *yellow* aerobic and *green* recovery. The *solid black line* in the top plot is the best fit for the mean within each segment. (Color figure online)

This example looks at detecting changes in heart-rate over a long undulating run. We use CROPS with ED-PELT with $\xi _{min} = 25$, $\xi _{max} = 200$ and $K = 4\log (n)$ (the results are similar for different *K*). In order to choose the best segmentation we use the approach suggested by Lavielle (2005). This involves plotting the segmentation cost against the number of changepoints and then looking for an “elbow” in the plot. The points on the “elbow” are then suggested to be the most feasible segmentations. The intuition for this method is that as more true changepoints are detected the cost will decrease however as we detect more changepoints we are likely to be detecting false positives and as such the cost will not decrease as much. The plot of the “elbow” for this example can be seen in Fig. 3a. The elbow is not always obvious therefore the choice can be subjective, in high throughput situations you can often learn a good choice of penalty through comparing segmentations for a range of training data sets (see Hocking et al. (2013)). However in this example the elbow approach this gives us a method for roughly choosing the best segmentations which we can then explore further. We have highlighted the points on the “elbow” as the points which are between the two red lines.

We decided from this plot that the segmentations with 9, 10, 12 and 13 changepoints are the best. We illustrate the segmentation with 10 changepoints, the number of changepoints at the centre of the elbow in Fig. 3a indicated by the blue circle, in Fig. 4. The segments have been colour coded based on the average heart-rate in each segment. That is red: peak, orange: anaerobic, yellow: aerobic and green: recovery. Alternative segmentations from the number of changepoints on the elbow can be found in the supplementary material.

We superimpose the changepoints detected in the heart-rate onto the plots for speed and elevation to see if we can explain any of the changepoints. The first segment captures the “warm-up” where the heart-rate is on average in the recovery zone but is rising to the anaerobic zone. The heart-rate in the second segment is in the anaerobic zone but changes to the peak zone in segment three. This change initially corresponds to an increase in speed and then it is because of the steep incline. The third changepoint matches up to the top of the elevation which is the start of the fourth segment where the heart-rate drops into the anaerobic zone whilst running downhill. The fifth segment is red which might be as a result of both the speed being slightly higher than the previous segment and consistent, and a slight incline in elevation. This is followed by a brief time in the aerobic zone which could be due to a drop in speed. The heart-rate in the next three segments stays in the anaerobic zone. The changepoints that split this section into three segments relate to the dip in speed around 75 min. In the final segment the heart-rate is in the peak zone which corresponds to an increase in elevation and an increase in speed (a sprint finish). We believe ED-PELT has found sensible segmentations that can be related to different phases of the run and regimes in heart-rate activity despite the data not satisfying the independence assumption.

### Piece-wise linear model

For comparison we look at estimating the changepoints based on a penalised likelihood approach that assumes the data is normally distributed with a mean that is piecewise linear within each segment. To find the best segmentation we use PELT with a segment cost proportional to minus the log-likelihood of our model:

5.1 $$\begin{aligned} \mathcal {C}(y_{s:t})=min_{\theta _1,\theta _2} \sum _{u=s}^t (y_u-\theta _1-u\theta _2)^2, \end{aligned}$$

where $\theta _1$ and $\theta _2$ and the estimates of the segment intercept and slope, respectively. We use CROPS to find the best segmentation under this criteria for a range of penalties. The resulting elbow plot can be seen in Fig. 3b. We can see that the number of changepoints for the feasible segmentations is similar to the number of changepoints for using ED-PELT. Figure 5 shows the segmentation with 9 changepoints which we have deduced to being the number of changepoints in the centre of the elbow in Fig. 3b. Alternative segmentations from the number of changepoints on the elbow can be found in the supplementary material.

It is obvious from the first look at Fig. 5 that the change in slope method has not detected segments where the average heart-rate is different to the surrounding segments. The majority of the plot is coloured orange with only changes in the first and last segments. The change in slope method splits the “warm-up” period into two segments whereas having this as one segment appears more appropriate. Unlike ED-PELT the change in slope does not detect changes which correspond to the change in elevation and thus ED-PELT appears to split the heart-rate data into more appropriate segments which relate to different phases of the run.

## Conclusion

We have developed a new algorithm, ED-PELT, to detect changes in data series where we do not know the underlying distribution. Our method is an adaption of the NMCD method proposed by Zou et al. (2014) which defines the segment costs of a data series based on the cumulative empirical distribution function and then uses an exact search algorithm to detect changes. The main advantage of ED-PELT over NMCD is that it is orders of magnitude faster. We initially reduced the time to calculate the cost of a segment from $\mathcal {O}(n)$ to $\mathcal {O}(\log n)$ by simplifying the definition of the segment cost by discrete approximation. To improve the computational cost Zou et al. (2014) use a screening step but as we show in Sect. 4 this is still slower than ED-PELT and less accurate. The main reason for this is we use an exact search algorithm, PELT, (Killick et al. 2012) that uses inequality based pruning to reduce the number of calculations. This search algorithm is much quicker than the one used in Zou et al. (2014).

The main problem with PELT is it requires a penalty value to avoid under/over-fitting and the performance is detrimental to this choice. We overcome this problem by using CROPS (Haynes et al. 2016), which detects the changepoints for multiple penalty values over a continuous range. Future research could look at an alternative pruning method, cp3o, proposed by James and Matteson (2015) which used probabilistic pruning. This method doesn’t require a penalty value however there are some mild conditions required for this search method which would need to be checked with the empirical distribution cost function.

We have also shown that nonparametric changepoint detection, using ED-PELT, holds promise for segmenting data from activity trackers. We applied our method to heart-rate data recorded during a run. As is common for current nonparametric approaches to changepoint detection, our method is based on the assumption that the data is independent and identically distributed within a segment. Despite this we were able to segment the data into meaningful segments, using an appropriately chosen penalty value, that correspond to different phases of the run and can be related to different regimes in heart-rate activity.

Code implementing ED-PELT is contained within the R library changepoint.np which is available on CRAN (Haynes 2016).

## Electronic supplementary material

Below is the link to the electronic supplementary material.

Supplementary material 1 (pdf 230 KB)
