# Supplementary material for: A computationally efficient nonparametric approach for changepoint detection
Source: Stat Comput. 2016 Jul 28;27(5):1293–305. doi: 10.1007/s11222-016-9687-5 (PMC6994226; doi:10.1007/s11222-016-9687-5)
Supplement: Supplementary file 1 — Supplementary material 1 (pdf 230 KB) [file 11222_2016_9687_MOESM1_ESM.pdf]

SUPPLEMENTARY MATERIAL

Further Results - NP-PELT+

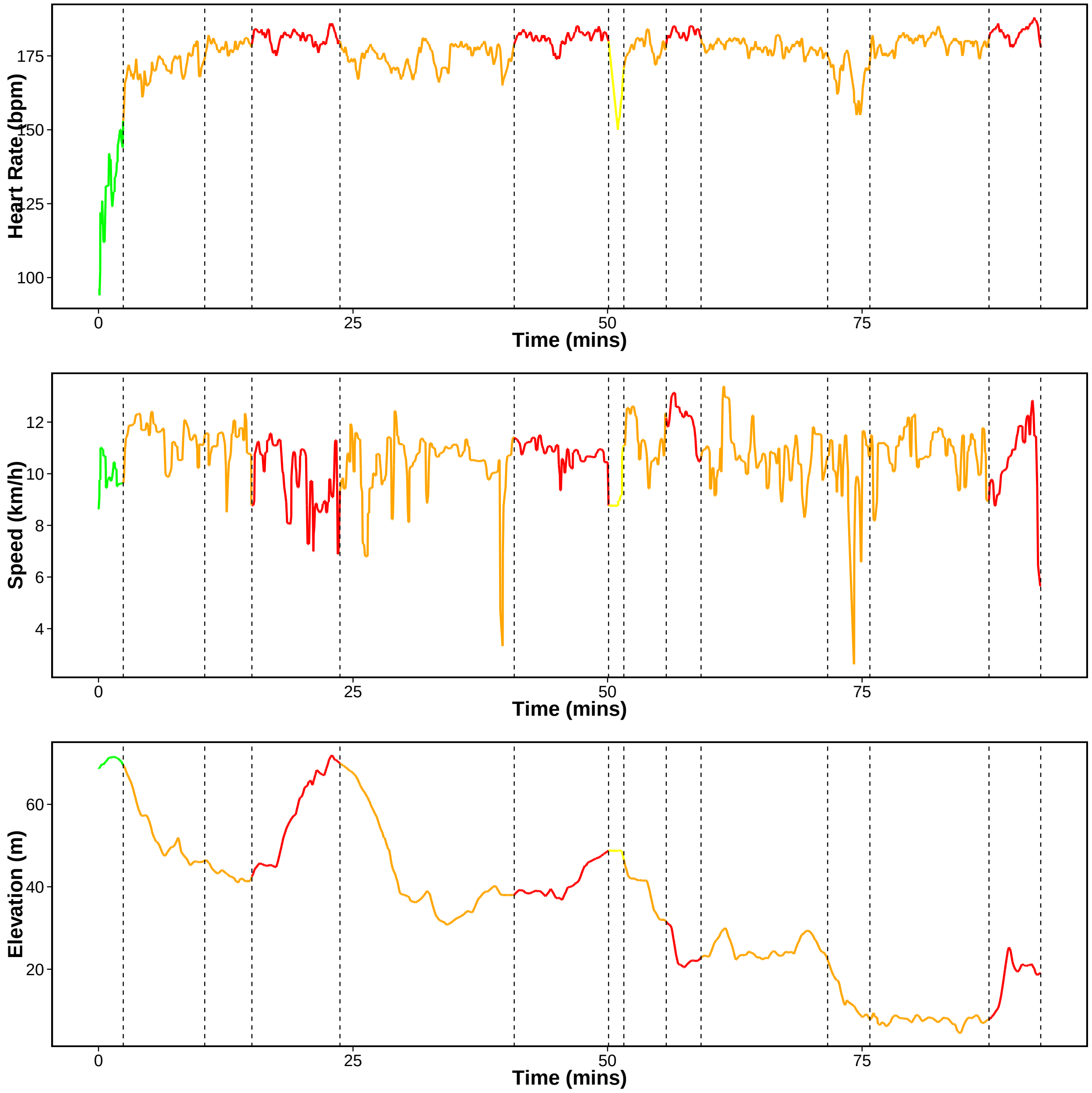

Figure 1: Segmentations using NP-PELT+ with 13 changepoints. We have colour coded the line based on the average heart-rate of each segment where red: peak, orange: anaerobic, yellow: aerobic and green: recovery.

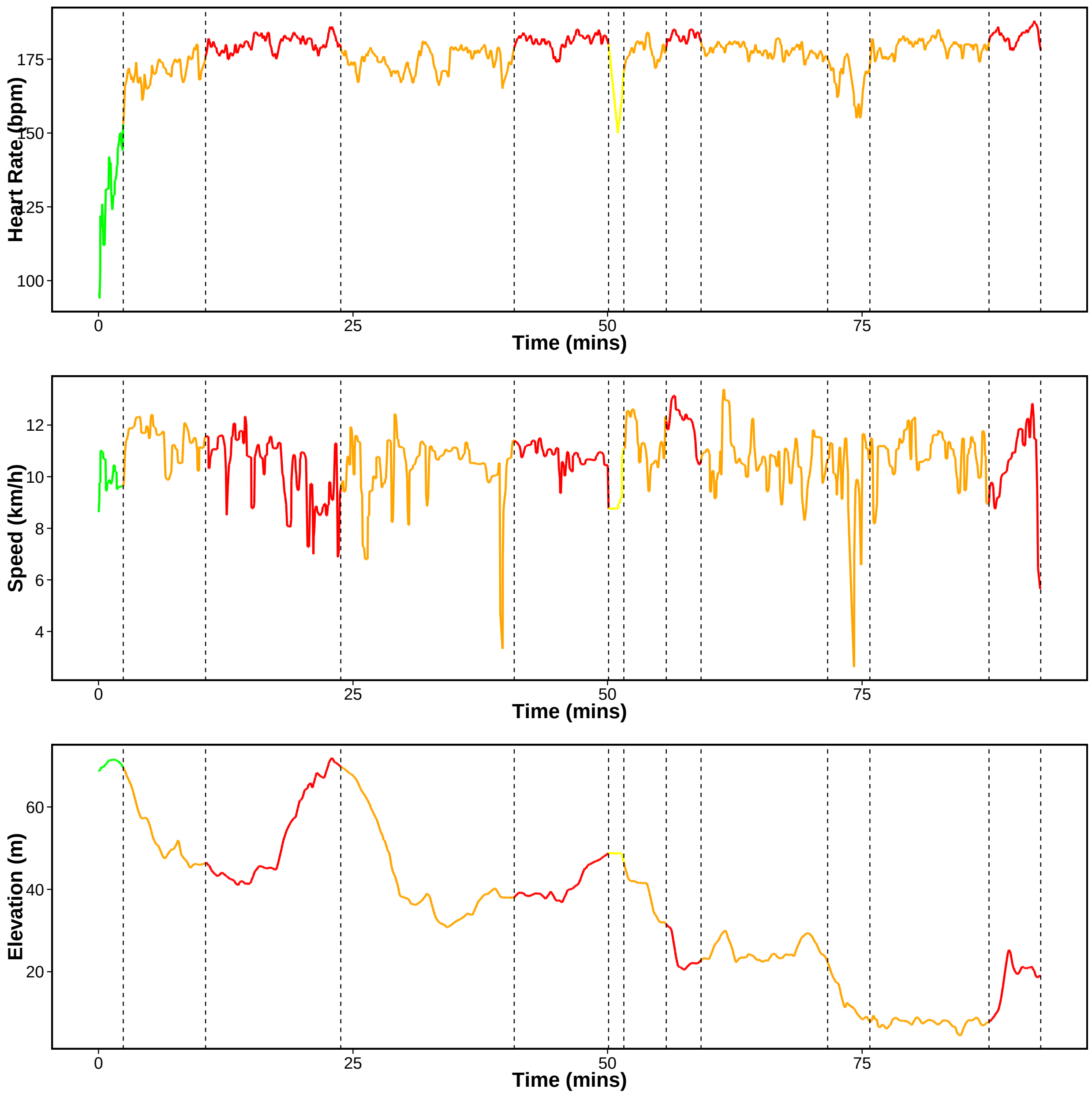

Figure 2: Segmentations using NP-PELT+ with 12 changepoints. We have colour coded the line based on the average heart-rate of each segment where red: peak, orange: anaerobic, yellow: aerobic and green: recovery.

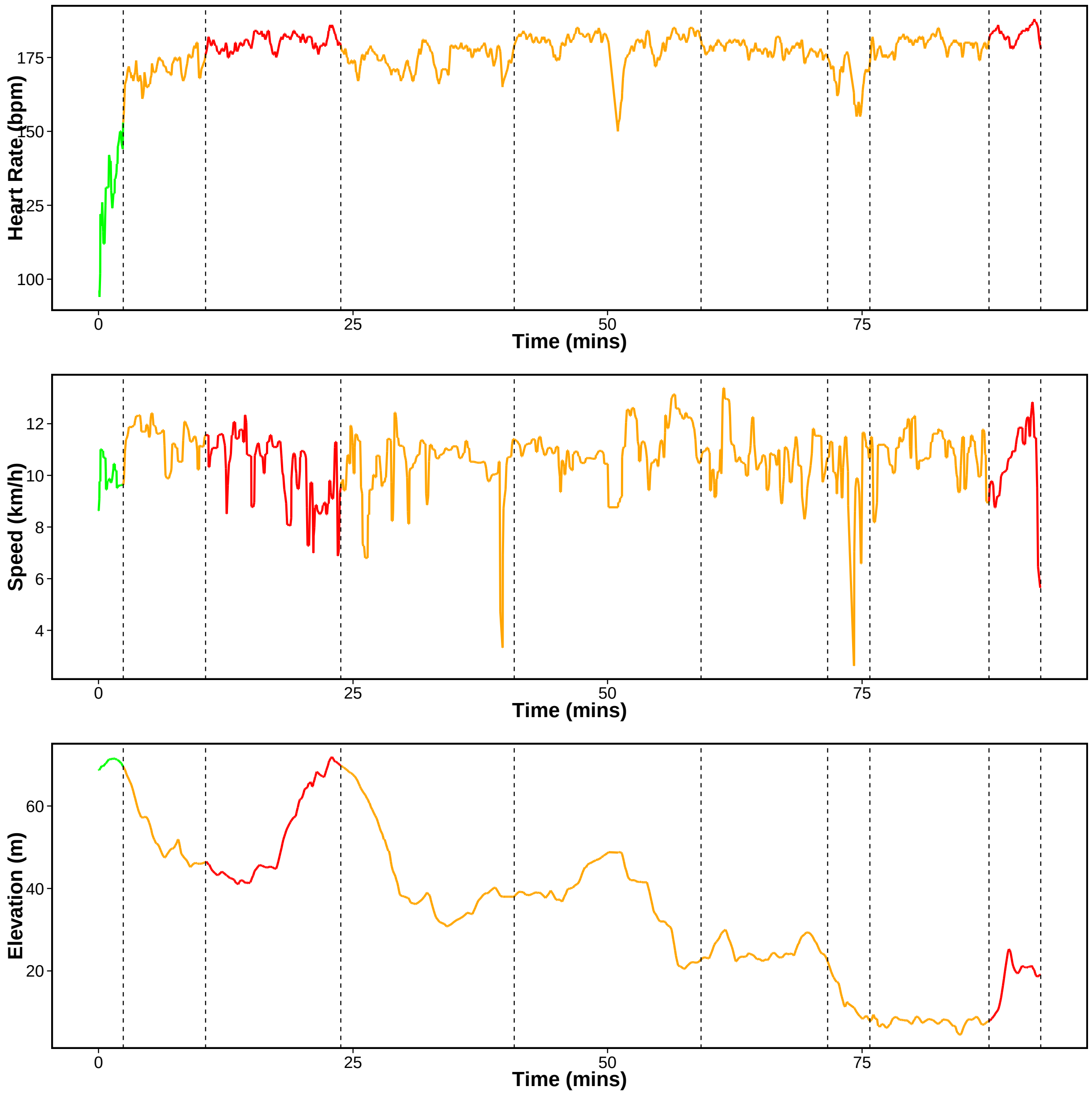

Figure 3: Segmentations using NP-PELT+ with 9 changepoints. We have colour coded the line based on the average heart-rate of each segment where red: peak, orange: anaerobic, yellow: aerobic and green: recovery.

Further Results - Piece-wise linear

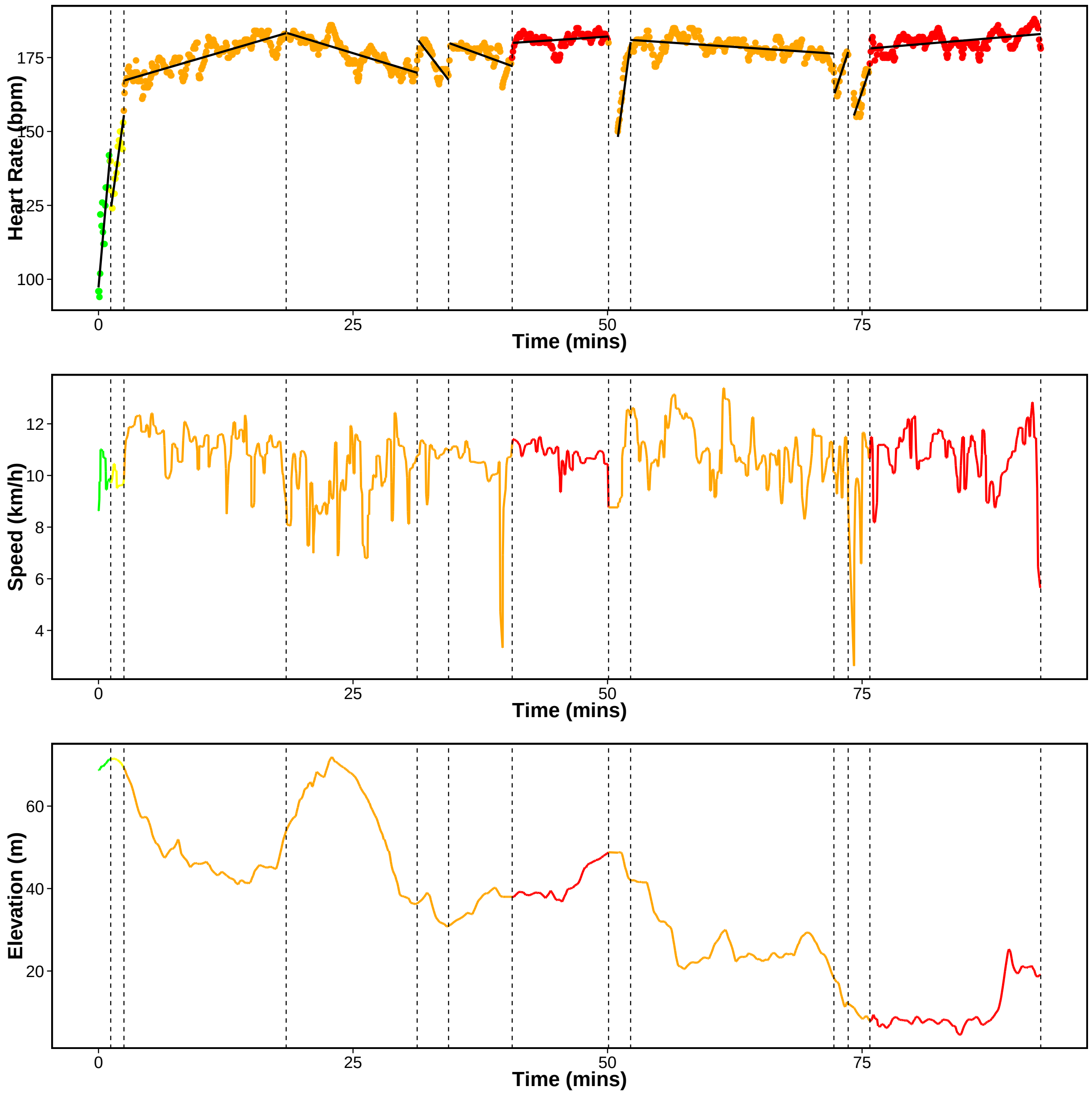

Figure 4: Segmentations using change in slope with 12 changepoints. We have colour coded the line based on the average heart-rate of each segment where red: peak, orange: anaerobic, yellow: aerobic and green: recovery. The solid black line in the top plot is the best fit for the mean within each segment.

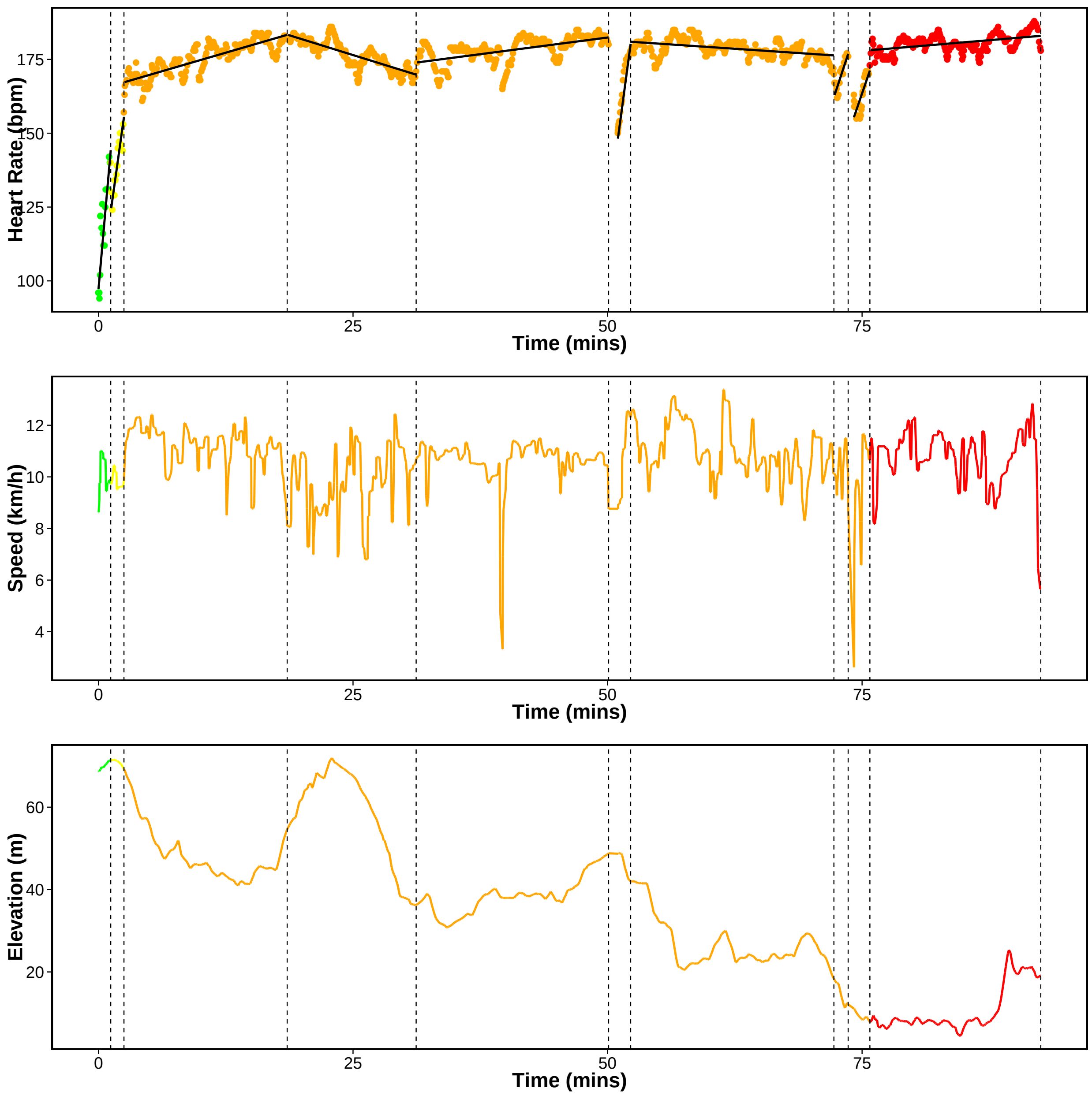

Figure 5: Segmentations using change in slope with 10 changepoints. We have colour coded the line based on the average heart-rate of each segment where red: peak, orange: anaerobic, yellow: aerobic and green: recovery. The solid black line in the top plot is the best fit for the mean within each segment.

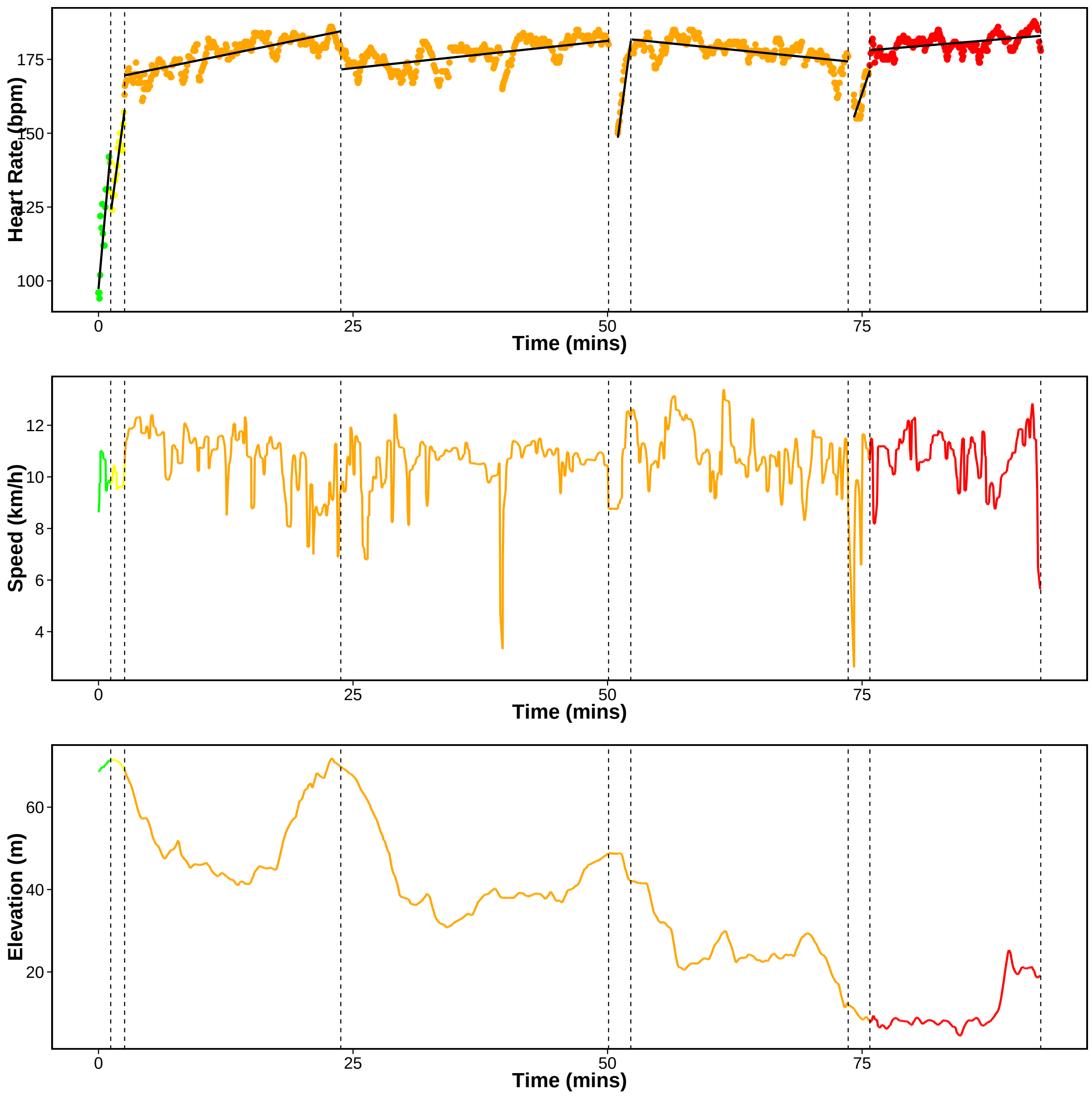

Figure 6: Segmentations using change in slope with 8 changepoints. We have colour coded the line based on the average heart-rate of each segment where red: peak, orange: anaerobic, yellow: aerobic and green: recovery. The solid black line in the top plot is the best fit for the mean within each segment.

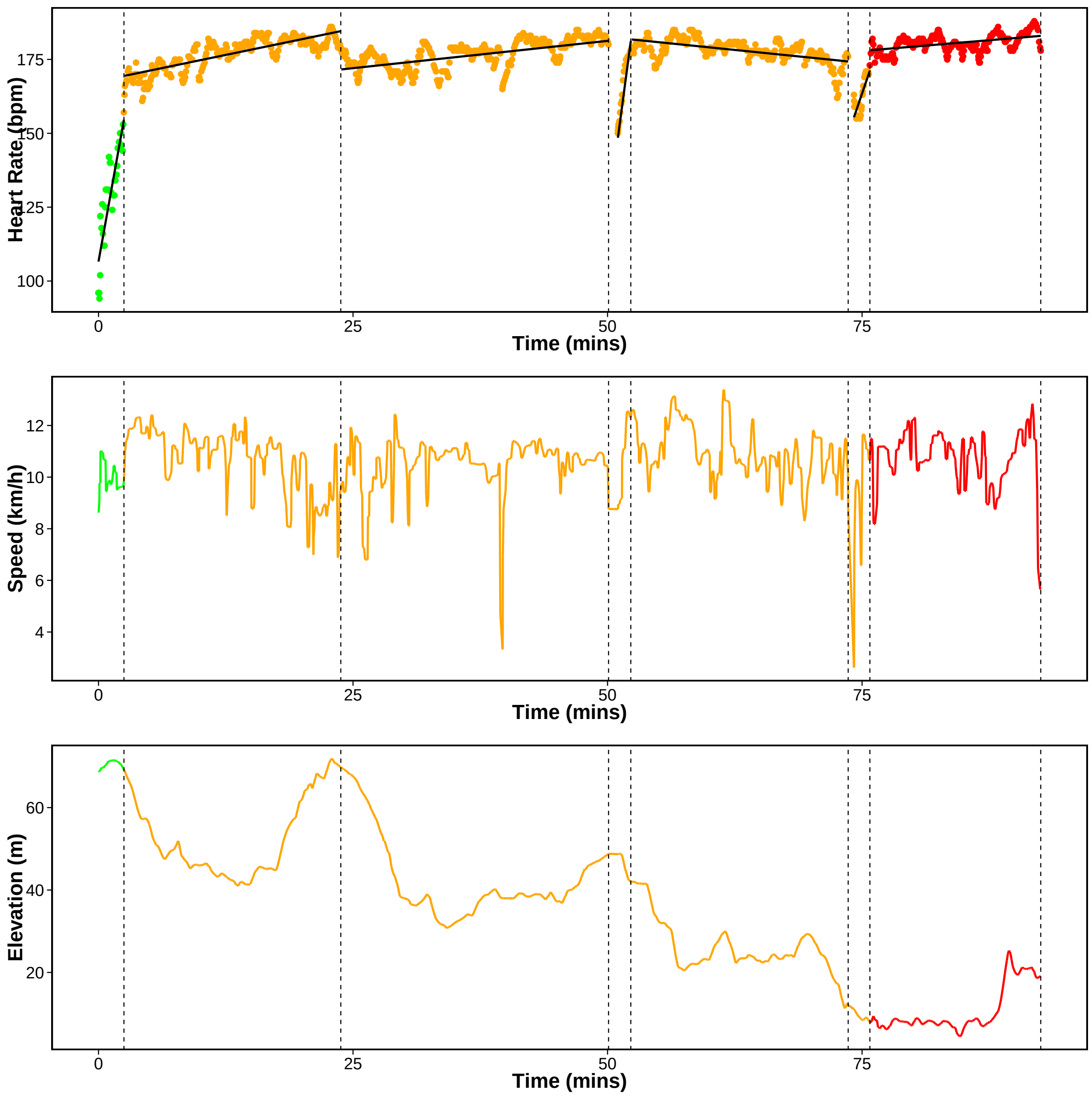

Figure 7: Segmentations using change in slope with 7 changepoints. We have colour coded the line based on the average heart-rate of each segment where red: peak, orange: anaerobic, yellow: aerobic and green: recovery. The solid black line in the top plot is the best fit for the mean within each segment.
